# Self-esteem and other risk factors for depressive symptoms among adolescents in United Arab Emirates

**DOI:** 10.1371/journal.pone.0227483

**Published:** 2020-01-14

**Authors:** Syed M. Shah, Fatima Al Dhaheri, Ammar Albanna, Najla Al Jaberi, Shaikha Al Eissaee, Nouf Ahmed Alshehhi, Shamma A. Al Shamisi, Maryam M. Al Hamez, Said Y. Abdelrazeq, Michal Grivna, Theresa S. Betancourt

**Affiliations:** 1 Institute of Public Health, College of Medicine & Health Sciences, United Arab Emirates University, Al Ain, UAE; 2 Boston Children’s Hospital, Harvard Medical School, Boston, Massachusetts, United States of America; 3 Child and Adolescent Mental Health Centre, Al Jalila Children’s Specialty Hospital, Dubai, UAE; 4 Division of Pediatric Rheumatology, Cincinnati Children’s Hospital Medical Center, Cincinnati, Ohio, United States of America; 5 Internal Medicine, Tawam Hospital, Al Ain, UAE; 6 Department of Epidemiology and Public Health, University, Ottawa, Ottawa, Canada; 7 Department of Global Health & Population, Harvard T.H. Chan School of Public Health, Boston, Massachusetts, United States of America; International Telematic University Uninettuno, ITALY

## Abstract

**Background:**

Little is known about depressive symptoms among adolescents in the United Arab Emirates (UAE). This study aimed to identify the prevalence of depression and its association with self-esteem, individual, parental and family factors among adolescents aged 12 to 18 in UAE.

**Methods:**

Six hundred adolescents, aged 12 to 18 years were recruited from 4 of 111 schools in a cross-sectional study. We administered Beck Depression Inventory Scale and Rosenberg Self-esteem Scale to measure self-report symptoms of depression and self-esteem. We used multiple linear regression to identify significant predictors of depression.

**Results:**

Over 86% of the identified sample participated to the survey. The mean age of the sample was 14.3 (±1.3) with an excess of girls (61%). Depressive symptoms were detected in 17.2% (95% CI 14.2–20.7). There was an inverse relationship between self-esteem scores and depressive symptoms. Positive predictors of depressive symptoms, having controlled for age, gender, and ethnicity included experiencing neglect, being verbally abused in school, having no monthly allowance to spend in school, a history of physical morbidities requiring treatment, being a current or past smoker and a low family income.

**Conclusion:**

The high prevalence of depressive symptoms measured in this survey suggests a significant public health problem among adolescents in the UAE. Public health interventions aimed at facilitating education and early detection and potential treatment of depressive symptoms are a priority in the region.

## Introduction

Childhood and adolescence is a critical time in the course of human development, beginning with the onset of sexual maturation and ending with the emergence of adult roles and responsibilities, influencing health and wellbeing that could last for a lifetime [1]. During this period major structural and connective development of the brain take place including the neural circuits and systems responsible for emotional regulation and complex reasoning. In this critical phase neurocircuitry is susceptible to the effect of the environment. A range of activities that adolescents undergo during this time have a significant impact on brain structure and function for years to come and through the entire lifespan [2, 3]. It is known that pre-pubertal boys are more likely than girls to experience depression. This trend is reversed during adolescence so that by the age of 15 years, females are almost twice as likely as males to experience an episode of major depression and this gender gap persists until age35-40 [4].

Depression is a common mental health problem in adolescents worldwide [5] and a major risk factor for complete suicide [6]. The consequences of depression include educational impairments, substance misuse, and cigarette smoking [7, 8, 9, 10]. Adolescent who experience symptoms of depression self-perceive their general health poorly, utilize health care services in excess and describe more negative work experiences compared to their non-depressed peers [11]. In children depression is particularly associated with poor social functioning, particularly with respect to peer relationships [12, 13].

Earlier on depression was considered an adult disorder and the diagnosis was based on the manifestation of core symptoms of persistent and pervasive sadness with loss of interest in pleasurable activities, low self-esteem, excessive guilt, suicidal thoughts, and changes in sleep and appetite (DSM-4) with little consideration to age-related changes [14]. Recent developments in neuroimaging and behavioral genetics provide the scientific base to understand the development of mental disorders and across the age span from childhood into adulthood [15]. These studies showed that anxiety and depression tend to vary phenotypically and genetically across development, and become more intertwined in adolescence [16]. Furthermore, depression rises dramatically with the transition from childhood through adolescence, with an estimated prevalence of 5% at preadolescence to 20% in young adulthood [17].

The prevalence of depression also varies by gender and levels of depression begin to rise more sharply in girls than boys in early teens [18]. Patterns of female depressive symptoms shift markedly at the onset of adolescents and the prevalence of depression becomes over two-fold higher in females during mid-teens as compared to their male counterparts and the early adolescent rise in female depression largely accounts for the persisting higher rates of depression in women than in men [19]. According to follow-up studies in US and Australian youth aged 10 to 15 years, social adversity around puberty predicted the persistence of depressive symptoms but did account for a pubertal rise in female depression [20].

According to a systematic review and meta-analysis of longitudinal studies [21], modifiable factors in adolescence that influence the risk of developing depression include substance use (alcohol, tobacco, cannabis and other illicit drugs), dieting, the insurgence of negative coping strategies, and protective life choices that favor healthy diets and regular sleep patterns. According to the longitudinal BELLA study [22], reports of mental health problem in parents predicted depressive symptoms in children and adolescents. Child reported that protective factors included self-efficacy, a positive family climate, and social support. All of these were associated with the development of less depressive symptoms over time. Family functioning has not only a significant role in the development of depression and anxiety, it remains an important risk factor for the onset of a wide range of difficulties in adolescence including eating disorders, and substance use [23, 24, 25].

Traumatic events can predispose adolescents to develop symptoms of depression and/or other psychiatric disorders. For instance motor vehicle accidents can induce maladaptive behaviors in children including a disturbed eating patterns which might be a reflection of psychological difficulties and emotional dysregulation [26, 27].

Burden and consequences of child maltreatment remains a major public health problem in high-income countries, as well as in developing countries with serious consequences to mental health, suicide attempt, and mortality in children and adolescents [28, 29]. Prospective studies indicate that children and adolescents exposed to emotional, neglect and other forms of maltreatment are more likely to develop depression [30, 31]. In these studies childhood maltreatment was specifically associated with depression relapse and reduced cortical surface brain area [32].

The burden of mental disorders in the Eastern Mediterranean region is higher than global levels, and depressive disorders were the third leading cause of non-fatal burden, according to the ‘Global Burden of Disease’ study [33]. There are only a few studies available in the United Arab Emirates to document prevalence of depression among adolescents aged 12 to 18 years and its correlation with self-esteem, child maltreatment, and other child and family factors. Early studies evaluated relationship between eating behavior, body size and depressive symptoms [34] and the prevalence of depression in adults and University students [35]. Only one study was available to document depressive symptoms in teen girls [36]. Therefore, the aim of the present study is to gain insight into the prevalence of depression and its associated factors including self-esteem, maltreatment, child and family factors among adolescents aged 12 to 18 years in Al Ain, Abu-Dhabi region, United Arab Emirates.

## Materials and methods

### Ethics statement

The study protocol was approved by the Al Ain Medical District Human Research Ethic Committee. Additional approval was also obtained from the Abu Dhabi Education Council’s Research Committee in order to conduct the study in schools. Parents were informed about the study though an information sheet describing the study. All the participants and their parents provided written informed consent.

### Study design and setting

This study adopted a cross-sectional design, which is the most appropriate epidemiological approach to identify the prevalence of symptoms of depression in any given population. The study was conducted in Al Ain, a fertile oasis city located approximately 160 kilometers east of the Abu Dhabi Capital. Abu Dhabi is the largest of the seven emirates that make up the United Arab Emirates.

### Participants

A multi-staged sampling technique was adopted to randomly select 4 schools from a sampling frame of 111 public and private schools enrolling children, aged 12 to 18 years in Al Ain, Abu Dhabi Emirates in UAE in 2015. The inclusion criteria included being a resident of Al Ain, UAE, age 12 to 18 years, male or female sex, ascent and parents’ signed informed consent (if aged 12 to 17 years), written informed consent of participant (if older than 17 years), absence of any serious illness. The sample size calculations were based on the intension to explore differences among subgroups. Assuming a conservative estimate of 12.5% prevalence of depression in UAE [35], a confidence level of 95% and a power of 80%, we would needed 250 participants per each group in a two-equal group comparison, with a total sample size of 500. We increased the target sample size to 600 to account for the possible refusal to participate. The targeted sample was selected with a probability proportional to the size of each school, while during the second-stage selection process, classes from each school were randomly selected by using the even and odd numbers of each grade. The targeted sample was selected with a probability proportional to the size of each school, while during the second-stage selection process, classes from each school were randomly selected by using the even and odd numbers of each grade.

### Instruments

An interviewer-administered questionnaire was developed and pilot-tested (n = 30) with information about socio-demographic variables (sex, age, grade, school attended and nationality), dietary habits, cigarette use, time spent in front of a screen playing video games, using computers and watching television. Other questions included previous and current health conditions, history of disease, required regular use of medication, and parents’ marital status and level of education. There were specific questions about maltreatment. Questions pertinent to sexual abuse were not approved by the Abu Dhabi Education Council due to cultural sensitivity. Included questions covered physical abuse (being pushed, grabbed, shoved, slapped, spanked, kicked, bit, punched, hit with on abject and choked) and emotional abuse (verbal abuse, threats of maltreatment, emotional neglect, feeling cared for, loved, social adversity around puberty predicted the persistence of depressive symptoms feeling comfortable, provision of food, clothes and medical care) [37].

#### Beck scale

Beck’s scale [38] is one of the most commonly used scale in the epidemiological studies to determine the prevalence of the symptoms of depression in population-based studies. There are a number of theoretical models to study depression in adolescents such as Beck’s cognitive theory of depression, and hopelessness theory of depression [39]. The Beck theory supports the hypothesis that loss, inadequacy, failure and worthlessness constitute the cognitive vulnerability. Beck Depression scale uses the Diagnostic and Statistical Manual of Mental Disorders (DSM-5) diagnostic system [40]. Depressive symptoms were evaluated with the 21-item Beck Depression Inventory (BDI-II), one of the most commonly used self-reported instruments to assess symptoms of depression [41]. Respondents were asked to rate the severity of each item on a scale from 0 to 3 based on their experiences in the previous week. The total scores ranged from 0 to 63. The scale has high internal consistency (a = 0.89). The BDI-II manual recommends that an index score of < 14 suggests no depression, 14 to 19 suggests mild depression, and ≥ 20 suggests moderate or severe depression. The BDI-II addresses all nine of the symptom criteria listed for a major depressive episode in the American Psychiatric Association’s Diagnostic and Statistical Manual of Mental Disorders. This scale has been validated in eighteen Arabic speaking countries including United Arab Emirates [42]. The BDI-II scale has a sensitivity of 96% and specificity of 63% at a cut-off of ≥18 scores [43].

#### Rosenberg Self-esteem scale

This scale is a self-report measure of self-esteem with high levels of reliability (internal consistency of 0.77 to 0.88) and it consists of ten statements focusing on general feeling toward the self [44]. Participants are asked to report grade of agreement in a four-point Likert scale (1 = agree not at all, 4 = agree completely). A higher score indicates positive self-esteem. This is the most widely used instrument for estimation of self-esteem including Arab and Emirati children [45, 46].

Trained school nurses distributed the self-administered questionnaire with Beck and Rosenberg self-esteem scale, and trained final-year medical students supervised the survey in schools and assisted with needed clarifications. Personal identification numbers were assigned to each adolescent participant to maintain anonymity. The identification number confirmed consent status and linked students to their respective schools. We used structured, self-administered questionnaires, available in both Arabic and English validated version, and participants completed either the English or the Arabic version. Investigators were available if respondents needed clarifications.

### Statistical analyses

All analyses were performed using STATA (Stata Corp LP 209), version 11.0. We described the socio-demographic characteristics of study adolescents, and characteristics of their parents. We used analysis of variance tests (ANOVA) and student t-test to explore whether there was a difference in the mean total score on BDI-II across the characteristics of adolescents and their parents. We used age as a categorical variable, gender and ethnicity as nominal variable, screen time as categorical variable, room sharing as binomial variable (yes, no type) monthly allowance as a categorical variable, cigarette smoking as binomial variable. We created the lowest, middle and the highest tertiles of the sum of self-esteem scores, and used it as categorical variable. We used types of abuse as binomial variable, history of disease as binomial variable, marital status as categorical variable, and monthly income as categorical variable. Statistical significance was defined as p-value of < 0.05. We used total scores on BDI-II as a continuous outcome variable for multivariable analyses to avoid power loss associated with categorization [47]. We included all significant variable in univariate analysis in a multiple regression model. We used multiple regression analysis to investigate the linear relationship between the response variable (depression score) and a number of predictors such as self-esteem, child factors, parental and family factors. Firstly, crude estimates (β) and 95% confidence intervals for each variable were calculated by using a uni-variable linear regression. We used multiple linear regression analyses to investigate significant predictors of depressive symptoms, by controlling for significant potential confounding variables such as age, income and ethnicity.

## Results

Out of 600 adolescents invited, 518 participated in this study (response rate 86.3%). The mean age of participants’ was14.3 (±1.3) years. Tables 1 and 2 describe the characteristics of the adolescents (1) and their parents (2). The age of participants ranged from 12 to 18 years and the majority were females (61.2%). Adolescents from Western and Arab countries showed significantly higher self-esteem scores compared to their counter-parts from United Arab Emirates and South Asia. The average self-esteem scores were 19.3, 20.1, 21.4 and 21.6 for South Asians, Emirati, Arab and Western adolescents respectively. A significant proportion of adolescents reported verbal abuse (34.0%), physical abuse (12.6%), and feelings of emotional neglect (12.1%). Overall, 82 adolescents (15.8%) reported a history of a disease that required medications. The top three reported diseases included asthma, anemia and allergy (Table 1). The vast majority of adolescents had both parents living in a monogamous family (94%). About half of the mothers were Emirati (46%), the other 42% were Arab mothers, and while very few (12%) were from other nationalities (Table 2).

**Table 1:** Characteristics of adolescents 12 to 18 years of age (n = 518)in Al Ain, United Arab Emirates.

Characteristic	n (%)
Age in years, mean (±SD)	14 (±1.8)
Gender Boys Girls	201 (38.8) 317 (61.2)
Ethnicity Emirati Other Arabs Westerners Asians	245 (47.4) 201 (39.0) 47 (9.0) 24 (4.6)
Daily hours spent on screen in the past 7 days < 1 hour 1 to 2 hours > 2 hours	51 (34.2) 46 (30.9) 52 (34.9)
Share room with others No Yes	204 (39.6) 311 (60.4)
Monthly allowance (1 AED = 3.7 USD) No monthly allowance < 500 AED 500–1000 AED > 1000 AED	123 (24.0) 221 (43.2) 122 (23.8) 46 (9.0)
Ever smoked cigarettes	62 (12.6)
Score on Rosenberg Self-esteem Scale, mean (±SD)	8.1 (± 7.1)
Lowest tertile of self-esteem score	76 (14.7)
Middle tertile of self-esteem score	201 (38.8)
Highest tertile of self-esteem score	241 (46.5)
Had been feeling emotionally neglected	62 (12.1)
Had been verbally abused in school	174 (34.0)
Had been physically abused (bullied) in school	65 (12.6)
History of disease that required use of medications	82 (15.8)
Asthma	23 (28.1)
Anemia	21 (25.6)
Allergy	14 (17.1)
Others	24 (29.3)
Score on Beck Depression Inventory (BDI) scale, mean (±SD)	20.9 (± 4.5)
No depressive symptoms (0–13 points)	429 (82.8)
Mild depressive symptoms (14–19 points)	47 (9.1)
Moderate to severe depressive symptoms (≥ 20 points)	42 (8.1)

**Table 2:** Characteristics of adolescents’ parents in Al Ain, United Arab Emirates.

Characteristic	N (%)
Marital Status of Parents Married Divorced Widowed Monogamous Polygamous	488 (94.6) 19 (3.7) 9 (1.7) 480 (93.0) 36 (7.0)
Number of Family Members 1–5 6–10 > 10	157 (30.4) 322 (62.3) 38 (7.3)
Nationality of Mother Emirati Arab Other	236 (45.7) 216 (41.9) 64 (12.4)
Father education No formal Up to secondary More than secondary	45 (12.3) 32 (8.7) 290 (79.0)
Mother education No formal Up to secondary More than secondary	45 (9.6) 107 (22.9) 315 (67.5)
Monthly family income (1 AED = 3.7 USD) < 10,000 AED 10,000–50,000 AED > 50,000 AED	79 (19.6) 210 (52.0) 115 (28.4)

**Fig 1:**
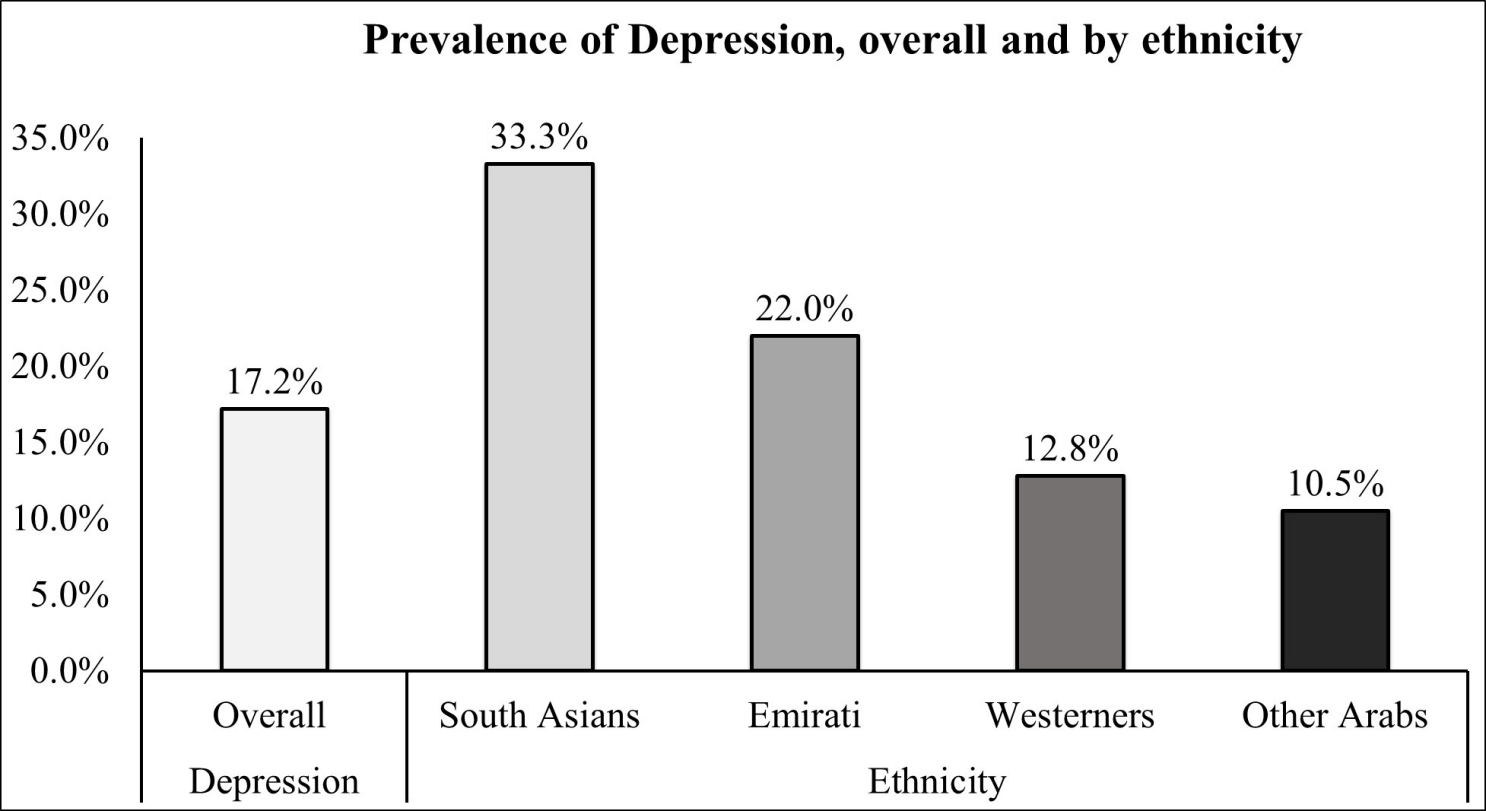
Prevalence of depressive symptoms among adolescents by ethnicity.

Overall, 89 out of 518 participants (17.2%; 95%CI 14.1–20.7) reported depressive symptoms using a cutoff score of ≥14 on the BDI-II scale. The prevalence of depression was high among girls (19.2%; 95%CI 15.0–24.0) compared to boys (13.9%; 95%CI 9.5–19.5), however this difference was not statistically significant (p value, 0.112). The majority of study participants (86.4%) were native Emirati or Arabs. Others belonged to Western (Australia, USA, and Canada) and South Asian countries. The highest prevalence of depression was noted among South Asian adolescents (33.3%; 95%CI 15.6–55.3) followed by native Emirati (22.0%; 95%CI 17.0–27.8), Westerners (12.7%; 95%CI 4.8–25.7) and Arab adolescents (10.4%; 95%CI 6.6–15.5) as shown in Fig 1.

The results of uni-variable analyses comparing the average the total Beck depression scale score across the groups of adolescents are described in Table 3. Having higher self-esteem was a significant and negative predictor of depressive symptoms. Those who were in the lowest quartile of self-esteem score had the highest prevalence of symptoms of depression (50.9%). Additional statistically significant positive predictors of a higher Beck scale score were not receiving a monthly allowance from parents, use of tobacco, feeling emotionally neglected, being a victim of verbal or physical abuse in school, and being treated requiring treatment. Other predictors of depression scores included being a child from a single-parent family, the mother being Emirati, and coming from family with monthly household income of less than 10,000 Emirati Dirhams (AED).

**Table 3:** Predictors of depressive symptoms (β and95% confidence intervals) among adolescents aged 12–18 years (n = 518) in Al Ain, United Arab Emirates.

Characteristic	BDI scores ≥ 14 N (%)	BDI scores total		
		Mean (SD)	Crude β (95% CI)	p-value
Age (years)				
12–13	32 (15.4)	7.9 (6.8)	Ref	
14–15	28 (17.3)	7.6 (6.8)	-0.35 (-1.81 to 1.11)	0.637
16–17	23 (18.4)	8.7 (7.5)	0.77 (-0.7 to 2.35)	0.334
>17	6 (26.1)	9.8 (8.3)	1.79 (-1.26 to 4.85)	0.249
Gender				
Boys	28 (13.9)	7.5 (6.7)	Ref	
Girls	61 (19.2)	8.5 (7.3)	1.01 (-0.24 to 2.27)	0.112
Ethnicity				
Westerners	6 (12.8)	7.0 (5.0)	Ref	
Emirati	54 (22.0)	8.9 (7.5)	1.95 (-0.24 to 4.14)	0.081
Other Arabs	21 (10.4)	7.0 (6.5)	0.02 (-2.21 to 2.25)	0.984
Asians	8 (33.3)	11.2 (9.6)	4.19 (0.73 to 7.64)	0.018
Daily hours spent on screen in the past 7 days				
< 1 hour	10 (14.5)	6.3 (5.4)	Ref	
1 to 2 hours	16 (16.3)	6.2 (4.9)	-0.18 (-2.78 to 2.42)	0.892
> 2 hours	63(18.0)	9.4 (8.4)	3.09 (0.57 to 5.61)	0.017
Share room with others				
Yes	45 (14.5)	7.5 (7.0)	Ref	
No	44 (21.6)	9.1 (7.0)	1.60 (0.35 to 2.85)	0.012
Monthly allowance (1 AED = 3.7 USD)				
< 500 AED	27 (12.2)	6.7 (5.7)	Ref	
500–1,000 AED	20 (16.4)	8.2 (6.9)	1.48 (-0.07 to 3.02)	0.061
> 1,000 AED	13 (28.3)	10.4 (9.5)	3.75 (1.53 to 5.97)	0.001
No monthly allowance	27 (21.9)	9.8 (7.9)	3.10 (1.56 to 6.64)	0.000
Ever smoked cigarettes				
No	69 (15.3)	7.6 (6.5)	Ref	
Yes	20 (32.3)	12.3 (9.5)	4.64 (2.80 to 6.49)	0.000
Rosenberg Self-esteem scale				
Lowest tertiles	27 (50.9)	16.9 (10.6)	Ref	
Middle tertiles	31 (17.1)	8.6 (7.9)	-6.02 (-7.61 to -4.43)	0.000
Highest tertiles	30 (10.9)	6.1 (5.6)	-8.27 (-9.86 to -6.67)	0.000
Had been feeling emotionally neglected				
No	41 (12.1)	7.4 (6.6)	Ref	
Yes	47 (27.0)	13.3 (7.9)	5.91 (4.11 to 7.72)	0.000
Had been verbally abused in school				
No	41 (12.1)	6.9 (5.8)	Ref	
Yes	47 (27.0)	10.7 (8.6)	3.82 (2.55 to 5.08)	0.000
Had been physically abused (bullied) in school				
No	73 (16.1)	7.8 (7.0)	Ref	
Yes	16 (24.6)	10.3 (7.2)	2.48 (0.65 to 4.32)	0.008
History of disease that required use of medications				
No	70 (16.1)	7.8 (7.0)	Ref	
Yes	19 (23.2)	9.7 (7.1)	1.86 (0.19 to 3.53)	0.029
Marital status of parents				
Parents married	77 (15.8)	7.9 (6.8)	Ref	
Parents divorced	7 (36.8)	12.4 (10.9)	4.53 (1.31 to 7.75)	0.006
One of the parent widowed	4 (44.4)	13.3 (5.2)	5.44 (0.81 to 10.08)	0.021
Nationality of mother				
Arab	24 (11.1)	7.1 (6.2)	Ref	
Emirati	52 (22.0)	8.9 (7.6)	1.78 (0.47 to 3.08)	0.008
Other	13 (20.3)	8.9 (7.5)	1.81 (-0.16 to 3.77)	0.072
Monthly family income (1 AED = 3.7 USD)				
AED > 50,000	21 (8.7)	7.4 (5.2)	Ref.	
AED 10,000–50,000	35 (17.4)	7.7 (6.5)	0.34 (-1.39 to 2.09)	0.696
AED < 10,000	33 (43.4)	10.0 (7.9)	2.64 (0.70 to 4.57)	0.008

Table 4 shows results of multiple linear regression analyses. Compared to those in the lowest tertiles of self-esteem scores, those in the middle or highest tertiles of the self-esteem scale were statistically significant negative predictors of Beck depression scale overall score. Statistically significant positive predictors of depression scores on the Beck scale included neglect, being verbally abused in school, having no monthly allowance to spend in school, a history of co-morbidities requiring medication, being a current or past smoker, and having a total family income of less than 10,000 Emirati Dirham, after controlling for age, gender, and ethnicity.

**Table 4:** Predictors of depressive symptoms among adolescents aged 12–18 years (n = 518) in Al Ain UAE*.

Variable	Adjusted β (95% CI)	p-value
Score on Rosenberg Self-esteem scale		
In middle tertile	-5.75 (-7.43 to -4.08)	0.000
In the highest tertile	-7.73 (-9.42 to -6.03)	0.000
Monthly allowance in Dirhams (1 AED = 3.7 USD)		
500–1,000 AED	0.41 (-1.05 to 1.88)	0.578
> 1000 AED	1.22 (-0.96 to 3.41)	0.271
No monthly allowance	3.37 (1.69 to 5.05)	0.013
Current or past cigarette smoker	3.37 (1.69 to 5.04)	0.000
Had been feeling emotionally neglected	3.17 (1.39 to 4.95)	0.001
Had been verbally abused in school	2.03 (0.82 to 3.25)	0.001
Had been physically abused in school	0.48 (-1.21 to 2.18)	0.573
History of disease that required use of medications	2. 51 (1.01 to 4.02)	0.001
Monthly family income < 10,000 AED	2.32 (0.59 to 4.06)	0.009
Monthly family income 10,000 to 50,000 AED	0.98 (-0.51 to 2.47)	0.196

* Multi-variable linear regression model adjusted also for age, gender, and ethnicity.

## Discussion

In this epidemiological survey, we set out to investigate the prevalence of depressive symptoms in a sample of adolescents from Al Ain in the United Arab Emirates. We found a relatively high prevalence of symptoms of depression (17.2%) in a school-based sample of adolescents representing over 86% of the local school population aged between 12 and 18 years. The prevalence of depressive symptoms noted in native Emirati and Arab adolescents falls within the range of previous studies in Arab world ranging from 5% to 30% [48], however, the prevalence is relatively higher compared to depressive symptomatology in developed countries, ranging from 4.0 to 8.2% [49, 50]. These variations may be attributed to the cultural differences and study populations, data collection tools used, as well as to the methodological issues of sampling processes, and various cut-off points of the used scale [51, 52].

In the present study the prevalence of depression varied across the many nationalities living in Al Ain. South Asian and Emirati adolescents had the highest prevalence of depressive symptoms with 1 in every 3 South Asians and 1 in every 5 Emiratis. The disparity in the prevalence of depressive symptoms in the South Asian group is of concern as this group with other migrants account for 80% of the total population of the United Arab Emirates [53]. High prevalence of depression and suicidal ideation was noted among South Asian adults in a previous study [54]. We also found that the prevalence of depressive symptoms reported by Westerners was much lower than Emirati adolescents (12.8% versus 22.0%). It is however noteworthy that this prevalence is higher than the reported estimates for developed countries, ranging from 4.0% to 8.2% [49, 50]. It would be of interest in future studies to investigate whether this increase reflects the psychological effect of some of the challenges faced by these young migrants when moving to the UAE or whether these symptoms or a degree of biological vulnerability were already present prior to leaving their native countries. A higher rate of depressive disorders has been documented in migrants with an aggregate prevalence of approximately 15.6% in the adult population [55]. It is also been shown that migrating mothers have an increased risk of conceiving a child with an autism-spectrum disorder compared with local mothers suggesting that at least for some diseases the possibility of gene x environment interactions [56]. In China, children of parents who migrated from rural to urban areas displayed greater mental health problems than expected in comparison with local children, especially in case of stressors within the family (e.g. single-parent families) and financial difficulties (e.g. low family income) [57].

In this work adolescents who had a more robust self-esteem reported lower scores on the Beck scale. Conversely, by using multivariable linear regression models we were able to identify several predictors of depressive symptoms. These included adolescents’ less disposable monthly allowance, more cigarette smoking, feelings of being neglected, or being abused in school and having a lower monthly family income.

We noted a higher prevalence of depressive symptoms among girls compared to boys. This finding is in agreement with other studies [58, 59]. This finding could be attributed to post-pubertal period as the advancing pubertal stage carried higher risks for depressive symptoms in females [20] associated with an enhanced social understanding, and increased stress levels [60]. A different style between boys and girls with regard to preferentially Internalizing or Externalizing behaviors as a way to react to emotional distress might also offer a putative explanation for our finding [61]. Internalization, more frequent in girls, refers to a tendency to withdrawing, experience somatic complaints, and symptoms of anxiety /depression. Externalization, more often associated with boys is more consistent with delinquent and aggressive behaviors. During adolescence, girls report higher psychopathological problems in areas consistent with ‘internalizing’ while boys have a tendency to externalizing. Parents, teachers and other care givers can play a mediating positive role during adolescence which can help alleviate stressors ultimately linked to the risk of developing significant psychopathology [62].

We noted that self-esteem scores were the lowest for the adolescents from South Asian countries whilst the prevalence of symptoms of depression was the highest (33.3%). High self-esteem is associated with individuals’ resilience and internal power, and is ultimately a reflection of the ability to cope effectively with adversity. Poor self-esteem was a strong, significant and independent predictor of depressive symptoms in our study in agreement with other studies [63].

In this study a sizeable number of study participants (15.8%) reported physical co-morbidities such as asthma, anemia and allergy. Such diseases are likely to interfere with many aspects of daily life, cause frustration and stress and in some instances can be directly linked with some of the symptoms of depression. They can also cause feelings of embarrassment, self-awareness and may interfere with school attendance and peer relationships. Previous studies have shown that depression is often associated with physical comorbidities [64]. Our study findings corroborates the need to screen children with physical co-morbidities for the presence of symptoms of depression. Prospective follow-up study have shown that increased levels of social and non-social stress and the presence of depression in adolescence impact later physical health in young adulthood and these effects persisted after controlling for sex [65, 66].

Usually parents give their children a regular amount of money to buy lunch from a school cafeteria and inculcate respect for the value and meaning of money. In our study, we found that many parents (76%) gave their children school allowance, however a sizable percentage of students reported not receiving any monthly allowance and this was a significant (p<0.05) predictor of high depression scores, although significance was not retained in the multi-variable adjusted model. Nevertheless, we also found that adolescents who were living in families with low household income had significantly higher depression scores and this was retained in the multi-variable model. Adolescents’ disposable allowance was highly correlated with family income and this is probably the reason for not retaining significance due to collinearity. Other studies examining the association of depression with household income have also reported that low socioeconomic status is associated with depression [21, 67].

Additional characteristics of adolescents were also significant predictors of depressive symptoms in the multi-variable adjusted model including cigarette smoking. Smoking in adolescence could lead to depression or depressed adolescents may start smoking as a stress related coping strategy [68]. Many longitudinal studies have reported on the bidirectional relationship of smoking with depression [69, 70]. Problems of addictive behaviors in adolescents (including, alcohol misuse, and problematic internet use) can be considered maladaptive strategies used to cope with negative emotions, especially that interactions between behaviors and emotions are particularly pronounced in this agegroup. [71, 72].

Child abuse in its various forms (emotional, physical, and sexual) has been a major focus for studying its link to future psychopathology. Exposure to Adverse Childhood Experiences (ACE) such as abuse or neglect, household violence or parental divorce in early childhood increases the risk of mortality and different forms of morbidity including autoimmune, liver, coronary and pulmonary diseases [73]. Exposure to ACE during the developmental period may also lead to many mental health problems including depression and substance abuse [74, 75]. In our study, we found a significant link between emotional and verbal abuse and depression. Emotional abuse may be the most common yet under-reported form of abuse. Verbal abuse in school is part of the bullying phenomenon, in which being a victim predisposes to future development of internalizing problems including depression, anxiety and suicidal ideation [76].

It is important to acknowledge a number of potential limitations in this study. Firstly, unlike a cohort study we could not establish temporal relation between the exposure risk factors and the development of the outcome of interest. Our major purpose was to document the prevalence of depression in adolescents and a cross-sectional design was the most appropriate study type for the aim. Establishing the prevalence of depressive symptoms in this age group and its associated significant risk and protective factors is the first necessary stage to subsequently design specific studies, preferential longitudinal design, to test inherent hypotheses, which might confirm or refuse the findings emerging from this work. Secondly, the recruitment of our study sample from the city of Al Ain (Abu Dhabi Emirate) may not be representative of the entire population of United Arab Emirates (UAE). However, we would not expect the socioeconomic and lifestyle characteristics of the study population to differ from other Emirates of the UAE. Nonetheless, this study report on the prevalence of depressive symptoms and its associated factors among a representative sample of adolescents living in Al Ain, UAE.

## Conclusions

In conclusion, we have documented a relatively high proportion of adolescents with depressive symptoms (17.2%) among adolescents in the UAE with South Asians having the highest detection rate (33.3%). Self-esteem was significantly inversely associated with depressive symptomatology. Additional correlates of depressive symptoms in adolescents included smoking, emotional, physical and verbal abuse in school and history of diseases requiring pharmacological treatment. Lower family income was also a significant risk factor for depressive symptoms. Findings from this study suggests that it is advisable to screen adolescents for identifiable vulnerability factors and potentially associated depressive symptoms to facilitate primary prevention, early detection of symptoms and the delivery of effective interventions whenever indicated. A synergic educational program at different community levels including schools and families is likely to improve primary and secondary prevention. The increase rate of newly arrived migrants appear to be susceptible to developing depression and it is imperative that more in the form of preventive strategies and increased assistance be incorporated to ensure their psychological wellbeing and improve their mental health outcomes. Further research should be conducted to better understand the risk of psychiatric disorders among members of this subpopulation.
